# Transcatheter percutaneous aortic valve implantation: The dream has become a reality

**DOI:** 10.4103/0256-4947.62824

**Published:** 2010

**Authors:** Walid M. Hassan

**Affiliations:** Consultant and Section Head of Adult Cardiology, Professor of Medicine, King Faisal Heart Institute MBC 16, PO Box 3354, Riyadh, Saudi Arabia hassanw@kfshrc.edu.sa

Aortic valve stenosis (AS) is increasing in frequency as the population ages.^1^ Severe AS is common, and when symptomatic, is associated with a predictably poor prognosis and high mortality.^2^,^3^ Although surgical aortic valve replacement (AVR) is still the gold standard for symptomatic patients with severe AS,^4^,^5^ in a sizable subset of high-risk patients the surgical option is excluded due to severe comorbidities and advanced age. In addition, several registries show that referring physicians often do not propose surgery; 33% of patients with severe symptomatic AS in the Euro Heart Survey were not being considered for surgery.^6^ Thus, despite the good results of valve surgery, there may well be a role for less invasive alternatives.

## Why Percutaneous Valve Implantation?

Balloon aortic valvuloplasty has a limited role in providing temporary relief of symptoms with high recurrence rates and a 1-year survival rate of only 54%.^7^ Its main role is palliation or as a bridge to surgical AVR. Medical therapy for these patients is associated with a dismal outcome.^8^ Thus, transcatheter percutaneous aortic valve implantation (TAVI) has recently emerged as an alternative to surgical AVR. This was first demonstrated in an animal model by Andersen et al in 1992, who delivered a porcine bioprosthesis attached to a wire-based stent at various aortic sites with satisfactory hemodynamic results.^9^

Since the first TAVI in a human in 2002,^10^ percutaneous heart valves (PHV) have already undergone several modifications from the first-generation devices. Currently, two PHV devices have been CE-marked (Conformité Européenne) and are in clinical application: a balloon-expandable and a self-expandable PHV. Despite initial skepticism, TAVI is becoming a clinical reality, with over 6000 procedures performed to date in many countries. Initial experience suggests that outcomes compare favorably with conventional valve surgery in selected patients. However, in the future a more mature procedure might represent a viable alternative for a much broader range of patients.

## Current Status

**Figure 1:**
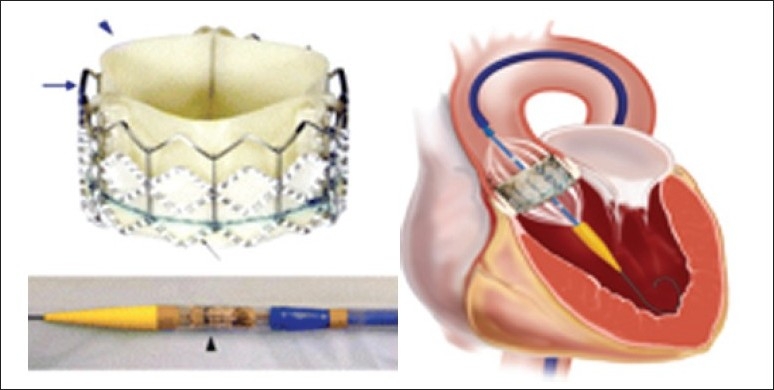
Edwards-Sapien percutaneous heart valves. Bovine pericardium leaflets (blue arrowhead) sutured (expanded polytetrafluoroethylene) (black arrow) onto a stainless steel stent frame (blue arrow) (Top). Delivery catheter with valve loaded (black arrowhead) (Bottom).

Currently, there are two PHVs in clinical trials, the balloon-expandable Cribier-Edwards (recently, the Edwards-Sapien) valve (Edwards Lifesciences Inc., Irvine, California), and the self-expandable CoreValve (CoreValve, Irvine, California). The balloon-expandable PHV consists of three pericardial leaflets, initially equine (Cribier-Edwards) and currently bovine (Edwards-Sapien), mounted within a tubular, slotted, stainless steel balloon-expandable stent (Figure 1). Current generation devices require either a 22-F or 24-F sheath for delivery.^11^ This PHV was initially implanted via the antegrade transseptal approach. There were several problems with this approach, and the retrograde approach has since been shown to be safer with the use of a proprietary steerable delivery catheter.

**Figure 2:**
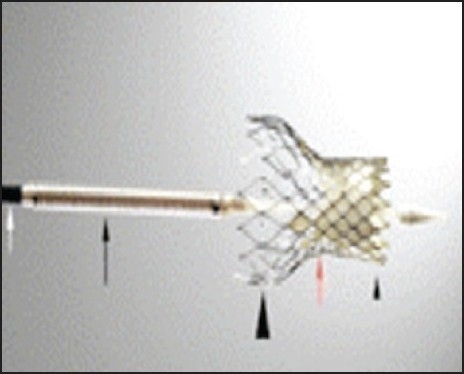
CoreValve PHV. A 12-F delivery catheter shaft (white arrow), 18-F delivery case, which contains valve (black arrow) with a flexible distal nose cone; opened valve consisting of the lower (inlet) portion (small arrowhead), the constrained middle portion to allow coronary perfusion (red arrow), and the upper (outlet) portion (large arrowhead).

The self-expandable PHV (CoreValve) consists of three pericardial tissue leaflets, initially bovine and currently porcine, mounted and sutured in a self-expandable nitinol stent (Figure 2). The stent frame is 50 mm, with the lower (inlet) portion having a high radial force to expand and exclude the calcified aortic leaflets; the middle portion carries the valve. The coaptation point of the leaflets is actually supra-annular-and is constrained to avoid obstructing the coronary arteries. The upper portion (outlet) is flared to fixate the stent in the ascending aorta and provide longitudinal stability. Early generation devices required 25-F sheaths; later devices incorporated porcine pericardial tissue constrained within 21-F, and now 18-F sheaths.^12^ Other valves and delivery systems with potential advantages in terms of the ability to deliver, deploy, or reposition the prosthesis are under development and in early pre-clinical evaluation.

## Indications

To date TAVI has been performed only in patients at high surgical risk. Objective estimates of surgical mortality have been used to help define “high-risk” like a logistic EuroSCORE >20 or Society of Thoracic Surgeons (STS) score >10. The other set of patients who may be considered at present are those with a deteriorated aortic bioprosthesis deemed at high risk for surgical reoperation, and this “valve-in-valve” concept has already been reported. With technological advancements, it is expected that the ease of implantation will improve and complications will decrease.

## Contraindications

The major specific contraindication to TAVI via the femoral artery is the presence of severe ilio-femoral stenosis. Some patients might have an aortic annulus that is too small or too large (current prostheses are suitable for an echocardiographic annulus diameter between 18 and 26 mm). Others might have an unusually bulky valve at risk of obstructing a coronary ostium. Mitral insufficiency and non-revascularized coronary disease are not necessarily contraindications; both are often well-tolerated in elderly patients, once aortic stenosis is relieved. However severe left ventricular dysfunction, severe mitral valve disease or nonrevascularized coronary disease can predispose to hemodynamic instability during TAVI.

## Procedure

The TAVI is typically performed in a cardiac catheterization laboratory or hybrid operating room. General anesthesia is optional. Femoral artery access and hemostasis might require a cutdown, but is increasingly accomplished with percutaneous closure. Conventional balloon valvuloplasty is initially performed. The valved stent is typically introduced into a femoral sheath and passed through the aorta. Positioning of the prosthesis within the native valve is confirmed with fluoroscopy, aortography, and often transesophageal echocardiography. Burst pacing might be used to transiently reduce transvalvular flow during deployment of balloon-expandable valves. The valved stent is expanded within the native aortic valve, displacing and excluding the diseased leaflets and substituting a new functional valve in place of the old. Discharge occurs as early as day 2, although median discharge might average 5 days as a consequence of delayed mobilization and disposition of elderly patients.

### Transapical Procedure

**Figure 3:**
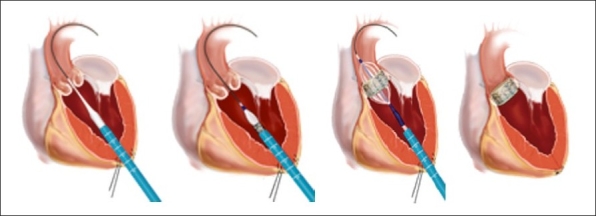
Transapical transcatheter percutaneous aortic valve implantation.

An alternative route of percutaneous valve implantation is via the open chest equivalent, which allows extension of the “transcatheter” technology to patients with vascular disease. This approach uses a mini-thoracotomy and needle puncture of the apical left ventricle without cardiopulmonary bypass (Figure 3). Reported experience is favorable, and as of early 2010 approximately 2000 transapical valve implantations have been performed worldwide.

## Complications

Of great concern is the possibility of arterial dissection or perforation while manipulating very large catheters through the vasculature. Myocardial ischemia or cardiogenic shock might occur and of concern is coronary obstruction by the prosthetic or native valve, although this seems infrequent. As with aortic valve surgery, TAVI is associated with a finite risk of atrioventricular block and pacemaker dependence. Reported rates of stroke with transcatheter valve implantation vary from 3% to as high as 9%.^13^ Hopefully these risks will diminish as the procedure improves and is applied to patients with less comorbidity.

## Outcomes

Initial transarterial experience in “high-risk” or “inoperable” patients was favorable, with successful implantation increasing from 76% to 96% with experience. Intraprocedural mortality was low at 2%. Mortality at 30 days after the procedure was 12%, comparing favorably to a logistic EuroSCORE estimate of 28% in this high-risk group.^11^

Overall, procedural success is closely linked to experience and is about 90% in experienced centers. A learning curve can also be observed resulting in better patient selection and outcomes. Valve function is good with a final valve area ranging from 1.5 to 1.8 cm^2^.

Mortality at 30 days ranges from 5% to 18%. Acute myocardial infarction occurs in 2% to 11%. Coronary obstruction is rare (<1%). Mild-to-moderate aortic regurgitation, mostly paravalvular, is observed in about 50% of cases. However, the availability of larger prostheses and their more careful matching with the size of the aortic annulus led to a decrease in the incidence of severe aortic regurgitation to about 5%. Prosthesis embolization is rare, about 1%. Vascular complications, with an incidence ranging from 10% to 15%, remain a significant cause of mortality and morbidity. Stroke ranges from 3% to 9%. Finally, atrioventricular block occurs in 4% to 8%, necessitating pacemaker implantation in up to 24% with self-expandable devices.

Long-term results up to 3 years (though only 1 year in most studies) are reported in a limited number of patients. They show a survival rate of 70% to 80% with a significant improvement in clinical condition in most cases. Serial echocardiographic studies have consistently shown good prosthetic valve function with no structural deterioration of valve tissue.

### Valve Function

**Figure 4:**
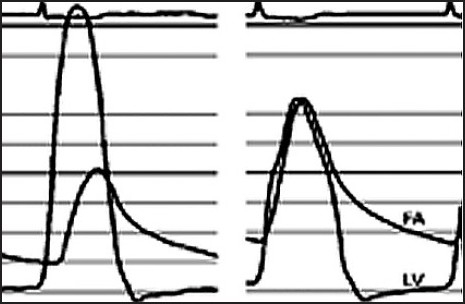
Transaortic gradients. Left ventricular (LV) to aortic gradients before and after percutaneous aortic valve replacement. FA=femoral artery.

In vitro and clinical evaluations of currently available transcatheter valves demonstrate excellent valve function (Figure 4). Orifice areas are typically larger than comparable surgical prostheses, owing to the absence of a bulky sewing ring and the ability to implant oversized prostheses after balloon dilation. Echocardiographic evaluation of both currently available valves typically documents gradients of fewer than 10 mm Hg and effective orifice areas of over 1.5 cm^2^. Although paravalvular regurgitation remains ubiquitous, such leaks are generally mild and hemolysis has not been observed.

### Late Outcomes

Acute and sustained improvements in left ventricular systolic function, functional mitral insufficiency, and functional class have been demonstrated after TAVI. In the high-risk candidates currently undergoing these procedures, late survival seems limited by comorbidities rather than valvular or coronary disease. Currently, on-going randomized controlled trials (PARTNER US and PARTNER EU) will help to determine the future of this revolutionary and promising interventional procedure. Physicians at King Faisal Heart Institute were among the first in the region to implement this sophisticated program with a very encouraging early result.

## Conclusions

The currently available results obtained with TAVI suggest that these techniques are feasible and provide hemodynamic and clinical improvement for up to 3 years in patients with severe symptomatic AS at high risk or with contraindications for surgery. Pending questions concern mainly safety and long-term durability. Surgeons and cardiologists must work as a team to select the best candidates, perform the procedure, and, finally, evaluate the results. Today, these techniques are targeted at high-risk patients but they may be extended to the lower risk groups in the future, if the initial promise holds true after careful evaluation. The road is long and arduous, but the dream has become a reality.
